# Proteomics-Based Evidence for a Pro-Oncogenic Role of ESRP1 in Human Colorectal Cancer Cells

**DOI:** 10.3390/ijms21020575

**Published:** 2020-01-16

**Authors:** Ugo Ala, Marta Manco, Giorgia Mandili, Emanuela Tolosano, Francesco Novelli, Paolo Provero, Fiorella Altruda, Sharmila Fagoonee

**Affiliations:** 1 Department of Veterinary Science, University of Turin, 10126 Turin, Italy; ugo.ala@unito.it; 2 Molecular Biotechnology Center, Department of Molecular Biotechnology and Health Sciences, University of Turin, 10126 Turin, Italy; marta.manco@unito.it (M.M.); emanuela.tolosano@unito.it (E.T.); paolo.provero@unito.it (P.P.); 3 Center for Experimental Research and Medical Studies, Azienda Universitaria Ospedaliera Città della Salute e della Scienza, 10126 Turin, Italy; giorgia.mandili@unito.it (G.M.); franco.novelli@unito.it (F.N.); 4 Center for Translational Genomics and Bioinformatics, San Raffaele Scientific Institute IRCCS, 20132 Milan, Italy; 5 Institute of Biostructure and Bioimaging, CNR c/o Molecular Biotechnology Centre, 10126 Turin, Italy

**Keywords:** ESRP1, proteomics, bioinformatics, colorectal cancer

## Abstract

The RNA-binding protein, Epithelial Splicing Regulatory Protein 1 (ESRP1) can promote or suppress tumorigenesis depending on the cell type and disease context. In colorectal cancer, we have previously shown that aberrantly high ESRP1 expression can drive tumor progression. In order to unveil the mechanisms by which ESRP1 can modulate cancer traits, we searched for proteins affected by modulation of *Esrp1* in two human colorectal cancer cell lines, HCA24 and COLO320DM, by proteomics analysis. Proteins hosted by endogenous ESRP1 ribonucleoprotein complex in HCA24 cells were also analyzed following RNA-immunoprecipitation. Proteomics data were complemented with bioinformatics approach to exploit publicly available data on protein-protein interaction (PPI). Gene Ontology was analysed to identify a common molecular signature possibly explaining the pro-tumorigenic role of ESRP1. Interestingly, proteins identified herein support a role for ESRP1 in response to external stimulus, regulation of cell cycle and hypoxia. Our data provide further insights into factors affected by and entwined with ESRP1 in colorectal cancer.

## 1. Introduction

RNA binding proteins (RBPs) exert a panoply of functions and regulatory interactions between RBPs, as well as with other components of the post-transcriptional control of gene expression occurring throughout all cellular processes [1]. RBPs may serve as a hub of signal integration by binding mRNAs available in a given cell type under a given condition, and hence perform a cell-type-specific function [2]. RBP-mRNA interaction, and through it, recruitment of other RBPs or classes of proteins, may regulate RNA turnover and determine cell fate. The aberrant expression of RBPs occurring in many human cancers with various underlying mechanisms, such as genetic alteration, epigenetic change, non-coding RNA-mediated regulation, and post-translational modifications, may amplify the effects of cancer-driver genes, accelerate tumor progression, and promote aggressiveness, hence highlighting the need for finely-tuned regulation of RBPs expression in the cell [3,4]. 

Epithelial Splicing Regulatory Protein (ESRP1) is an epithelial cell-specific RBP and splicing factor, which has been described as a tumor suppressor in several studies due to its role in negatively regulating Epithelial-to-Mesenchymal Transition (EMT), for instance, in breast and pancreatic cancer, in oral squamous cell and non-small cell lung carcinomas [5,6,7,8]. A pro-metastatic activity of ESRP1 has also emerged from more recent studies. Indeed ESRP1, by regulating the alternative splicing of its target, CD44, also promotes lung metastasis of 4T1 breast cancer cells as well as brain-metastatic progression of melanoma, with elevated ESRP1 expression negatively correlating with patient survival [9,10]. Jeong et al. have more recently demonstrated that ESRP1 has a cancer-promoting role in ovarian cancer [11]. We also showed that ESRP1 is important for the anchorage-independent growth of CRC cells and that high ESRP1 expression may thus stimulate growth of cancer epithelial cells and promote colorectal cancer progression, by stimulating the autocrine activation of the fibroblast growth factor receptor (FGFR1/2) pathway [2].

ESRP1 is also a key regulator of cellular protein expression either through the generation of alternative isoforms or through translation regulation. Leontieva et al. showed that ESRP1 is capable of binding the 5′UTR of mRNAs and induces alterations in protein levels of several cancer-related genes such a *c-myc* and *fos* [12]. Other potential target proteins of ESRP1 in pancreatic cancer include IQ motif containing GTPase activating protein 1, heat shock protein 70, vimentin and perilipin 3, for which no splicing variants exist [6]. Interestingly, transient transfection of ESRP1 in PANC-1 cells led to an increase in expression of proteins including α-enolase, which is a prognostic marker in patients with pancreatic cancer, and filamin-α, which interacts with many proteins related to cancer metastasis [13,14,15,16]. Of late, an increasing amount of studies have provided evidence of the occurrence of interactions between RBPs [1]. These proteins, through cooperation, antagonistic interaction or mutual heterogeneous/autogenous interactions, regulate common RNA targets, ultimately leading to the alteration in target protein levels [1]. However, the complete scenario of how ESRP1 promotes carcinogenesis in a subset of CRC patients still remains to be elucidated.

In the present study, we further investigated, at the molecular level, into the function of ESRP1 in promoting colon carcinogenesis. Our proteomics data generated on CRC cells modulated for ESRP1 expression, complemented with publicly available data on protein-protein interaction (PPI) and bioinformatics analysis, reveal new functions of ESRP1 in CRC. Moreover, we provide an insight into the candidate proteins assembled onto ESRP1 regulatory complex in CRC cells. Our data may facilitate the identification of novel ESRP1-driven therapeutic opportunities to selectively target cancer cells.

## 2. Results

### 2.1. Proteomics Analysis Reveals Differential Expression of Several Cancer-Related Proteins upon Modulation of ESRP1 Expression in Human CRC Cells

**Figure 1:**
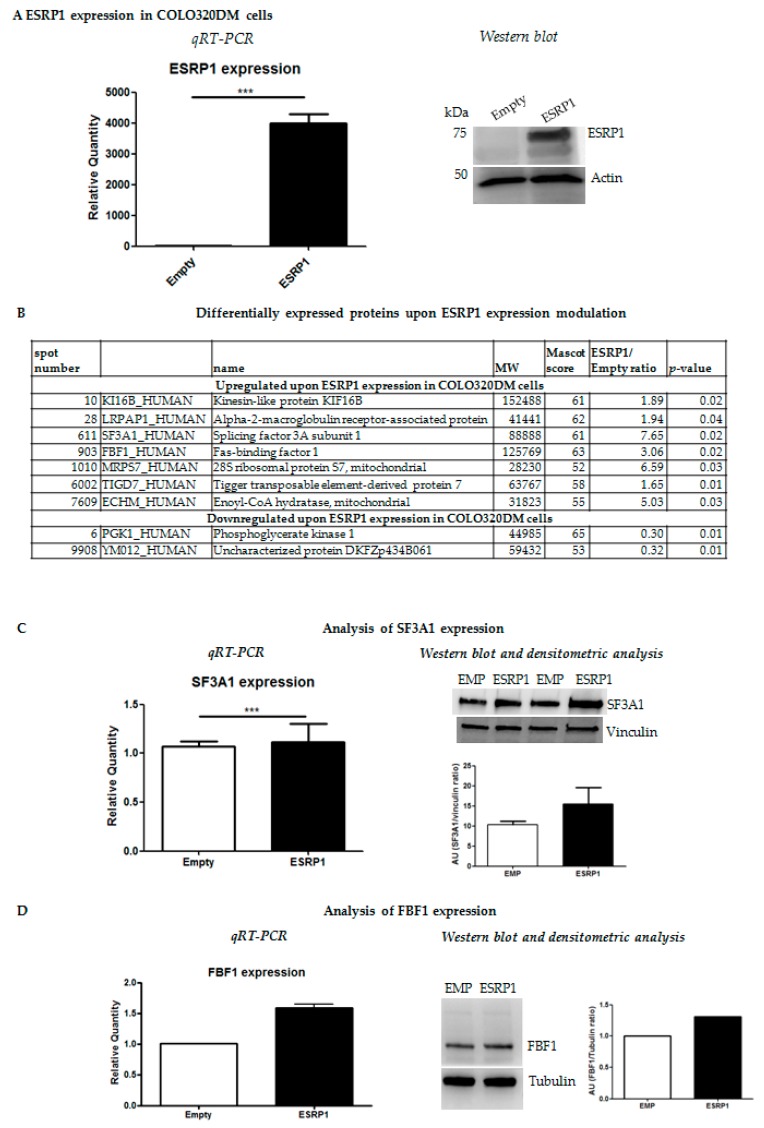
ESRP1 expression modulation in COLO320DM cells and proteomic analysis. (**A**). ESRP1 over-expression (ESRP1) in COLO320DM cells versus Empty controls (Empty) was analyzed by qRT-PCR and western blotting. (**B**). Proteins revealed as differentially expressed by MALDI-TOF analysis are shown. (**C**). Validation of results by qRT-PCR (*n* = 3) and western blotting/densitometric analysis (representative results of 2 independent experiments) of SF3A1 is shown. *** *p* < 0.0001 (**D**). Validation of results by qRT-PCR (*n* = 3) and western blotting/densitometric analysis (representative results) of FBF1 is shown.

**Figure 2:**
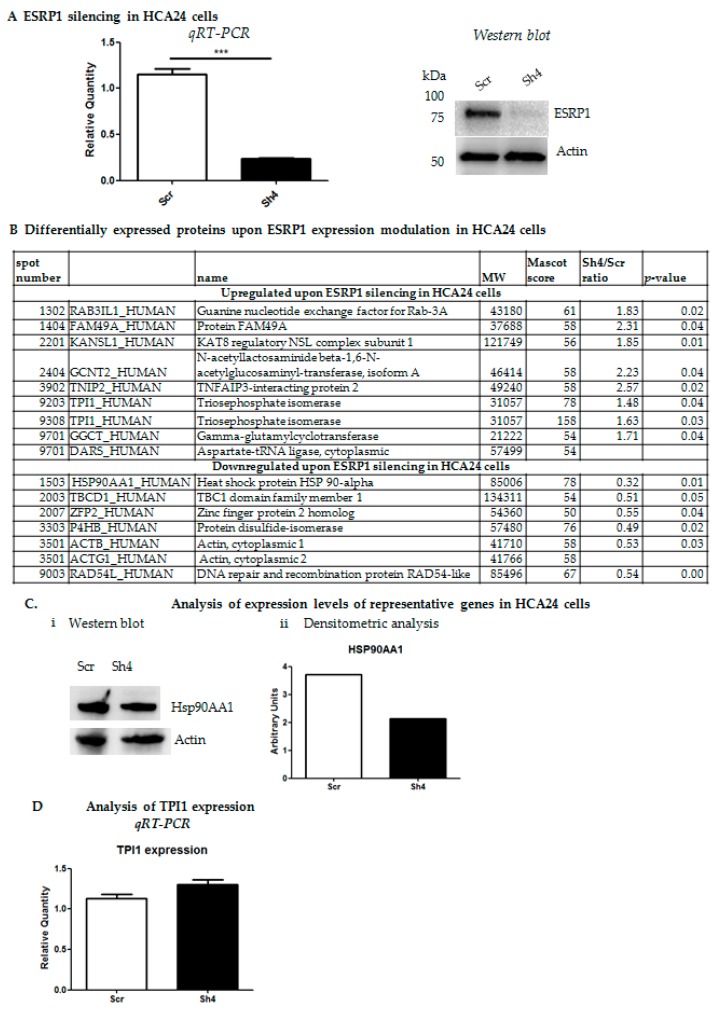
ESRP1 expression modulation in HCA24 cells and proteomic analysis. (**A**). ESRP1 silencing (Sh4) in HCA24 versus scrambled (Scr) controls was analyzed by qRT-PCR and western blotting. *** *p* < 0.0001. (**B**). Proteins revealed as differentially expressed by MALDI-TOF analysis are shown. (**C**). Validation of results by qRT-PCR (*n* = 3) and western blotting/densitometric analysis (representative results) for Hsp90AA1 is shown. (**D**). Validation of results by qRT-PCR (*n* = 3) for TPI1 is shown.

We previously showed that ESRP1 expression silencing in human HCA24 and Caco-2 cells reduced their tumorigenicity while overexpression of ESRP1 in Caco-2 cells promoted proliferation in anchorage-independency and transformation, and enhanced liver macro-metastasis formation of COLO320DM cells [2]. In the present work, we further characterized, at the molecular level, the ESRP1^low^ COLO320DM and ESRP1^high^ HCA24 cells undergoing *Esrp1* over-expression or silencing, respectively [2]. COLO320DM cells were infected with concentrated lentiviral particles over-expressing human ESRP1 ORF at a Multiplicity of Infection (MOI) of 1.5. Short hairpin RNA (Sh4)-mediated stable *Esrp1* silencing was performed in HCA24 cells using lentivirus at a MOI of 1. ESRP1 expression modulation in both cell lines was verified (Figure 1A and Figure 2A).

To determine the mechanistic consequences of *Esrp1*-overexpression or -silencing on protein expression in COLO320DM and HCA24 cells, respectively, we performed 2D gel electrophoresis (2-DE). Nine spots were identified in COLO320DM cells and 16 spots in HCA24 cells upon *Esrp1* modulation *versus* controls, and analysed by MALDI-TOF (Supplementary Materials). Over-expression of ESRP1 in COLO320DM cells resulted in an increase in seven proteins including alpha-2-macroglobulin-receptor-associated protein (LRPAP1), a multifunctional endocytic receptor recently identified as a hub within a biomarker network for multi-cancer clinical outcome prediction; splicing factor 3A subunit 1 (SF3A1), which may be up-regulated in head and neck cancers, rectal carcinomas, and human non-small and small-cell lung cancers; Fas-binding factor 1(FBF1) and Trigger transposable element-derived protein 7 (TIGD7) (Figure 1B) [17,18]. A decrease in two proteins was significant in this cell line following the expression in ESRP1 including phosphoglycerate kinase 1 (PGK1) a prognostic biomarker for cancer (Figure 1B) [19]. We chose to validate the ESRP1-induced differential expression of selected candidates by qRT-PCR and western blot, and confirmed the modulation of SF3A1 both at RNA and protein level in COLO320DM cells (Figure 1C), as well for FBF1, which showed a positive correlation with ESRP1(Figure 1D).

On the other hand, *Esrp1*-silenced HCA24 cells exhibited an increase in eight proteins including Guanine nucleotide exchange factor for Rab-3A (RAB3IL1); Protein FAM49A (FAM49A); N-acetyllactosaminide beta-1,6-N-acetylglucosaminyl-transferase, isoform A (GCNT2), which is induced upon EMT in colon cancer cells; TNFAIP3-interacting protein 2 (TNIP2), which may modulate the microenvironment for colorectal cancer development, and Triosephosphate Isomerase (TPI1) involved in glycolytic metabolism, and a decrease in seven proteins including heat shock protein (HSP) 90-alpha (HSP90AA1), which plays a fundamental role in several cellular processes pertaining to tumorigenesis such as cell proliferation and survival and malignancy such as invasion, angiogenesis and metastasis; Protein Disulfide-isomerase (P4HB) involved in the proliferation, survival, and metastasis of several types of cancer cells; Actin, cytoplasmic 1 (ACTB), a cytoskeletal protein, essential for the structure and kinetics of the cytoskeleton; and DNA repair and recombination protein RAD54-like (RAD54) involved in double-strand break repair (Figure 2B) [20,21,22,23,24]. Selected candidates were validated by qRT-PCR and western blot, and confirmed the down-regulation of HSP90AA1 at protein level (Figure 2C), as well as a slight increase inTPI1 at RNA level in HCA24 cells (Figure 2D). Overall, the expression trend, albeit subtle, of the candidate proteins selected for validation correlated with proteomics data.

The expression of selected candidates (SF3A1, TPI1 and FBF1) revealed by mass spectrometry from the two cell lines were further analysed in ESRP1-silenced or -overexpressing Caco-2 cells (Supplementary Materials) in order to assess whether the proteins identified in this study were cell line specific or part of a common pathway involving ESRP1. Interestingly, TPI1 and FBF1 showed differential expression upon ESRP1 modulation, but with an opposite trend with respect to that observed in COLO320DM or HCA24 cells, indicating that the changes in proteome were cell-specific.

#### Functional Characterization of Differentially Expressed Proteins Ranked Correlated Sets

Taking into account the highly context specific but relatively small amount of proteins identified, we mined the STRING protein-protein interaction (PPI) database in order to expand the number of proteins related to ESRP1 and its newly found co-players. A Ranked Correlated Set (RCS) was defined for each protein found associated to ESRP1 in HCA24 and COLO320DM cell lines upon ESRP1 expression modulation.

**Figure 3:**
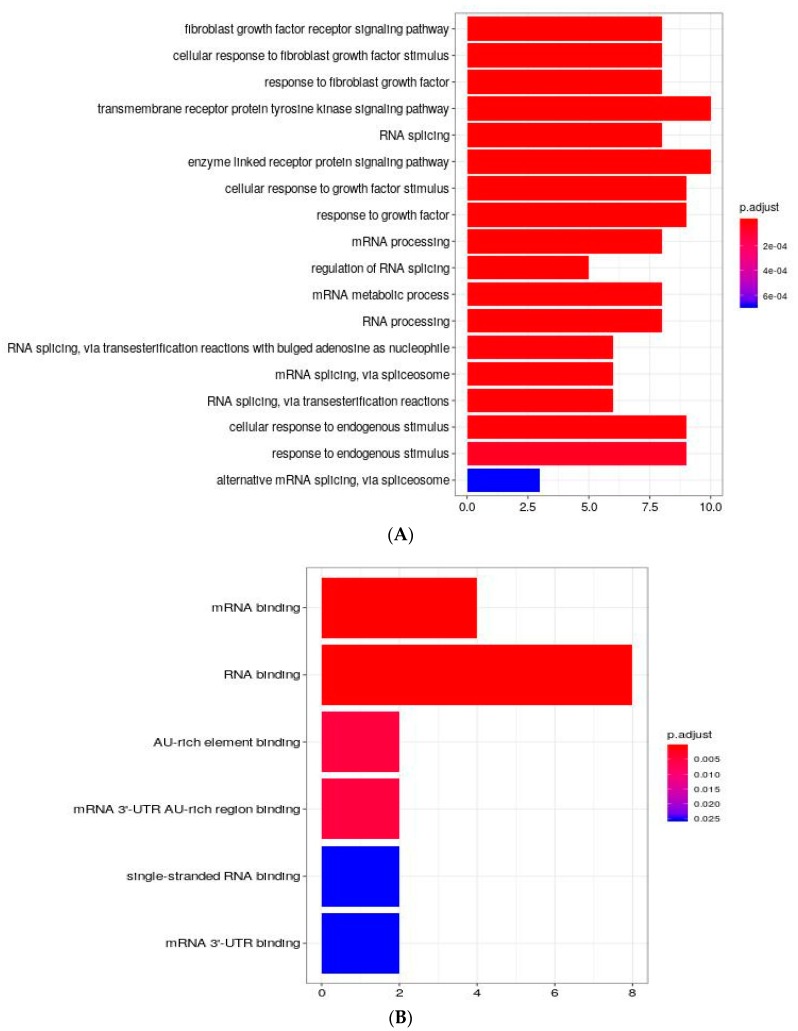
ESRP1 RCS Functional Characterization. (**A**) ESRP1 RCS Biological Process Enrichments; (**B**) ESRP1 RCS Molecular Function Enrichments; on the x-axis the number of proteins driving the significance is reported; the Benjamini-Hochberg method was used to correct *p values* for multiple comparisons.

Among the 24 proteins found with differential expression, we discarded FAM49A, TIGD7, YM012, ZFP2, and RAB3IL1 due to the lack of the minimum number of requested interacting proteins in STRING. The remaining 19 proteins were analyzed for molecular and functional characterizations by considering the GO and GSEA Hallmark enrichment analysis (see Materials and Methods section). Firstly, ESRP1 RCS was checked to see if ESRP1 characterization found in STRING was consistent with known information from literature on this protein. Several enriched GO terms associated with *RNA splicing* and *mRNA splicing* were still associated to this specific RCS showing that the number of proteins considered in RCSs was biologically informative, as a positive control (Figure 3A). Regarding new involvements, enrichments in GO Biological Process domain related to response to growth factor, fibroblast growth factor receptor signaling pathway, transmembrane receptor protein tyrosine kinase signaling pathway and alternative mRNA splicing, via spliceosome were revealed. With respect to Molecular functions, ESRP1 was found involved in *mRNA binding* and *RNA binding* (Figure 3B), confirming previous data [25,26]. ESRP1 positive control-similar keywords, like *regulation of RNA splicing* and *mRNA splicing*, via *spliceosome*, were found in other RCSs, such as HSP90AA1 and SF3A1, resulting, interestingly, from different lists of proteins, thus further strengthening these molecular involvements. Moreover, TNIP2 and LRPAP1 RCSs support new associations such as *response to growth factor* term, while HSP90AA1, DARS, MRPS7and SF3A1 RCSs underline the role in *RNA binding* molecular function. Other sets pertaining to differentially expressed proteins, like ACTB, ACTG1, TBC1D1 and P4HB RCSs, stressed different important aspects related to cell junction, in particular *adherens junction* and *anchoring junction*. In a more general way, enrichments appear in the following processes: *regulation of response to stress*, *organic cyclic compound compound process*, *nitrogen compound transport*, *hexose process*, *cell cycle*. The cell components more involved are linked to *melanosome* and *protein-DNA complex* and the molecular functions more involved are *protein kinase binding*, *monosaccharide binding*, *DNA-dependent ATPase activity* and *enhancer sequence-specific DNA-binding*.

### 2.2. Proteomic Analysis of ESRP1-Interaction Network in CRC Cells

**Figure 4:**
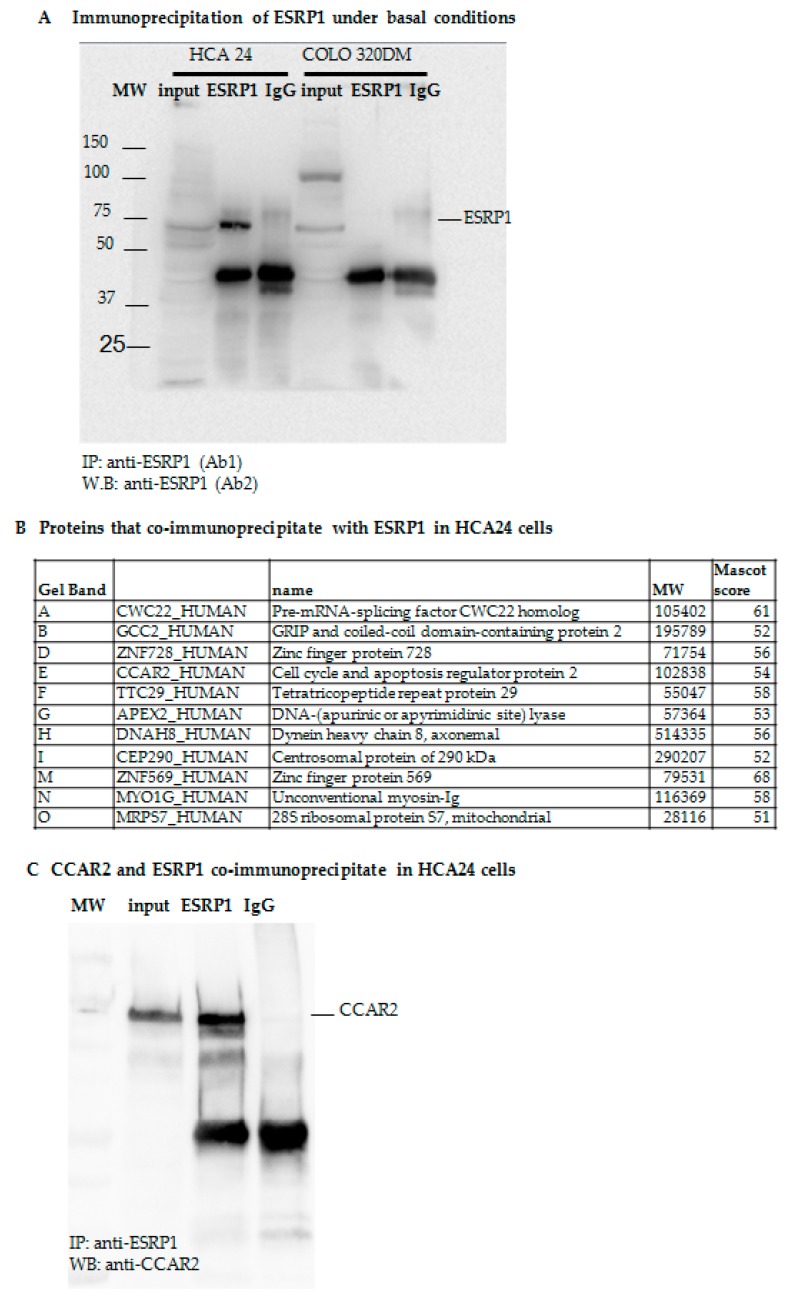
RIP-derived endogenous ESRP1 interactors in HCA24 cells. (**A**). Western blot showing ESRP1-antibody specificity in RIP. COLO320DM cells were used as negative controls. (**B**). Proteins co-immunoprecipitating with ESRP1 and identified by MALDI-TOF analysis are shown. (**C**). Validation of candidate ESRP1 interactor (CCAR2) by RIP and western blotting (representative of 3 independent experiments).

Proteins forming part of a complex have high probability of being functionally related [27]. In order to analyze proteins interacting with endogenous ESRP1 in CRC cells, we performed RNA-immunoprecipitation (RIP) using protein extracts obtained from ESRP1^high^ HCA24 cells under basal conditions (Figure 4A). Proteins immunoprecipitating with ESRP1 versus IgG were separated by 2-DE, and differential bands were selected (Supplementary Materials) and analyzed by MALDI-TOF. This analysis found 11 proteins immunoprecipitating with ESRP1, among which cell cycle and apoptosis regulator protein 2 (CCAR2 alias DBC1, KIAA1967) was an optimal candidate, which could partly explain how ESRP1 participated in cancer progression in CRC cells (Figure 4B). Results were validated using protein extracts obtained from HCA24 cells by RIP with anti-ESRP1 antibody or anti-IgG control followed by immunoblot with anti-CCAR2 antibody. CCAR2 was found to specifically co-immunoprecipitate with ESRP1 (Figure 4C).

In order to investigate whether ESRP1 can regulate the expression of CCAR2, we further analysed the expression of CCAR2 in HCA24 cells with modulated ESRP1 expression by real time-PCR, but did not find any significant correlation between the two proteins (Supplementary Materials). The CCAR2 level remained unchanged also in COLO320DM cells with differential expression of ESRP1 (Supplementary Materials). Moreover, in Caco-2 cells, no significant difference in CCAR2 expression upon silencing or over-expressing ESRP1 was found (Supplementary Materials), suggesting that CCAR2 is an interactor of ESRP1 but its expression is not regulated by the latter.

#### ESRP1-Interaction Network Ranked Correlated Sets Functional Characterization

As done previously for differentially expressed proteins, the RCSs of the 11 proteins found immunoprecipitated with ESRP1 were studied. Due to the small number of interacting proteins in STRING, we discarded MYOG1, TTC29, ZNF728 and ZNF569. The remaining 7 RCSs, through for instance CCAR2 and CWC22, showed a convergent ESRP1 behavior linking further *RNA splicing* and *response to growth factor* related keywords, such as *regulation of cellular response to growth factor stimulus*. Interestingly, the RCSs of CCAR2, CWC22 and MRPS7 were characterized by *RNA binding activity*. CCAR2 and CEP290 RCSs further underlined the cell junction involvement through *cell-cell junction, adherens junction* and *anchoring junction*. The overall RIP proteins trend of enrichments was based on regulation of *protein modification process*, *cell projection organization*, *carbohydrate derivative binding*, *enzyme binding* and *spindle*.

### 2.3. Global Tendencies of Enrichments

In order to enhance the biological information obtained from the functional analysis, we pulled all the significantly associated terms belonging to all RCSs, independently, thus obtaining the most frequently enriched GO keywords (Supplementary Materials). In particular, regarding biological process, the most enriched terms were related to *regulation of protein ubiquitination*, *nucleoside trisphophate metabolism*, *DNA duplex unwinding* and *chordate embryonic development*. Molecular functions were more related to *molecular binding and molecular activity*, such as *kinase binding, enhancer binding, ATP binding, ATPase activity* and *protein kinase activity*. Focusing on the terms potentially pertaining to cancer and different from the enrichments related to ESRP1 characterization already discussed, we highlighted other interesting findings like *involvement in regulation of cell proliferation* in CCAR2 and TNIP2 RCSs; Mitosis through *regulation of mitotic cell cycle* in CCAR2, CEP290, DNAH8, FBF1, HSP90AA1 and RAD54L RCSs and through *microtubule cytoskeleton organization* involved in mitosis in CCAR2, DNAH8, KIF16B and CEP290 RCSs; *G2*/*M transition* evidenced from CCAR2, CEP290, DNAH8, FBF1, TBC1D1 and RAD54L RCSs; Translation in CCAR2, DARS, MRPS7, P4HB and HSP90AA1 RCSs; Transcription for regulation of DNA-binding transcription factor activity in CCAR2, TNIP2 and LRPAP1 and posttranscriptional regulation of gene expression in HSP90AA1, CCAR2 and TNIP2 RCSs. Interestingly, all these biological features are highlighted by RCSs belonging to proteins derived from both experimental strategies used to find new ESRP1 co-players (Supplementary Materials). Among the different GSEA hallmark gene sets, the term hypoxia was found in the global enrichment pertaining to two RCSs, PGK1 and TPI1.

## 3. Discussion

An increasing number of studies describe ESRP1 as an oncogenic RBP [28,29]. In a recent review by Garcia-Cardenas et al. ESRP1 was described as one of the 35 RBPs (out of a total of 1393 RBPs) bearing an oncogenic role in CRC [30]. As ESRP1 shows a plastic behaviour during tumor development, proteins or genes regulated by ESRP1 are expected to undergo cell- and context-dependent modulation [7]. ESRP1 localisation may also be important in directing its function, for instance, alternative splicing of target genes in epithelial cells when ESRP1 is nuclear or controlling translation when ESRP1 is cytoplasmic.

To get further insights into pathways activated to sustain a pro-oncogenic function of this RBP, we performed proteomic analyses on CRC cells differentially expressing ESRP1. Apart from ESRP1, several proteins of the spliceosome have been implicated in cancer development [30]. The abnormal expression of splicing factors, including ESRP1 and SF3A1, are known to modify splice site selection, thus generating mRNA isoforms that contribute to the development, progression and therapeutic response of cancers [16]. From our proteomics dataset, SF3A1 emerges as a splicing factor positively correlated with ESRP1 expression in CRC cells. SF3A1 expression was found to be up-regulated in rectal carcinomas as well as in other cancers [18]. In order to correlate the data obtained by proteomics analysis to human clinical CRC samples, we furthered our analysis by analysing tumor samples from Wang et al. [31]. As seen in Supplementary Materials, correlation analysis shows that several of the proteins identified experimentally in our study exhibited statistical significance. In particular, SF3A1 and CCAR2 were significantly positively correlated to ESRP1 in the CRC patient samples considered.

**Figure 5:**
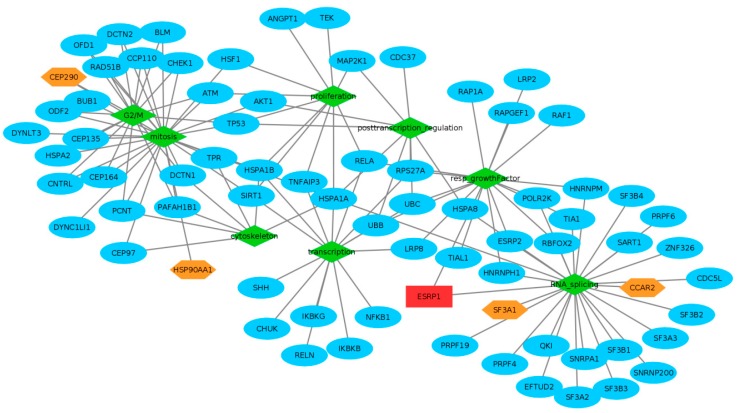
ESRP1 Extended Functional Network. Connections of some GO enriched keywords (depicted in green rhombus) and some ESRP1 partners highlight ESRP1 role and its extended biological influences from RNA splicing to response to growth factors, from mitosis and transcription to proliferation and post-transcriptional regulation. Edges connect GO terms with their associated proteins; red rectangle highlights ESRP1; orange hexagons proteins found experimentally by proteomics; blue ellipses proteins associated to our RCSs by STRING database.

As proteins belonging to the same protein complex are often functionally related, PPI analysis can provide further hints into interaction network and functions of a protein. ESRP1 is a 681 amino acid polypeptide that contains 3 RNA recognition motifs, a N-terminal predicted DnaQ-like exonuclease domain, and a proline-rich region that shares homology with DAZ-associated protein 2 (DAZAP2) [32]. This suggests that ESRP1, apart from recruiting proteins through RNA, may also be involved in protein-protein interactions that drive several intracellular signaling pathways. We analyzed, using RIP followed by proteomic-based identification of candidates, which proteins potentially associate with endogenous ESRP1 in CRC cells. We show herein that CCAR2 co-immunoprecipitates with ESRP1 in HCA24 cells, but its expression was not regulated by ESRP1 level in the different CRC cell lines used in the present study. CCAR2, found on human chromosome 8 (8p21.3), is involved in multiple cellular processes, and like ESRP1, it has a dual role in cancer depending on the context [33]. Importantly, CCAR2 is a core component of the DBIRD complex, a multiprotein complex, which acts at the interface between core messenger RNP particles and RNA polymerase II, and integrates transcript elongation with regulation of alternative splicing [34]. CCAR2 depletion could affect the anchorage-independent growth of several types of cancer cells [35]. Taking these data into account, and on the basis of our present data, we hypothesize that CCAR2, which is present at high levels even in ESRP1-negative cells such as COLO320DM, can perform its multiplicity of function, independent of ESRP1. On the other hand, ESRP1, when present, recruits CCAR2, either through direct binding of CCAR2 to ESRP1 (through its proline-rich region that shares homology with DAZ-associated protein 2 (DAZAP2), or through common RNA target binding, and participates in conferring an oncogenic function to ESRP1. Interestingly, the integrated approach we employed highlights a common involvement of SF3A1 and CCAR2 in specific Biological Processes where ESRP1 itself participates (Figure 5). SF3A1 is involved in pre-mRNA splicing, and is a component of the splicing factor SF3A complex that binds in a sequence-independent manner to anchor the U2 snRNP to pre-mRNA [36]. The aberrant expression of splicing factors, such as SF3A1, can modify splice site selection, and hence modulate alternative splicing of tumor promoters or suppressors, resulting in the formation of mRNA isoforms. This could contribute directly or indirectly to the development, progression and therapeutic response of cancers [18]. Our data also show that ESRP1 interacts and is co-modulated with a group of proteins that are strongly associated with a pro-mitotic and pro-proliferative activity. Notably, this set of proteins could guide in understanding the mechanisms underlying the pro-tumorigenic role of ESRP1 observed in CRC cell lines.

The functional characteristics of ESRP1 RCS highlight biological processes in which ESRP1 is potentially involved, such as RNA splicing and mRNA processing. The analysis also reveals that ESRP1 participates in response to growth factors. This is in line with our previous data showing that ESRP1 induces, through AKT1 activation, the release of FGFs in cancer cells, hence activating an autocrine signalling loop. Thus, data obtained using an independent approach confirms the role of ESRP1 in FGFR signalling. Figure 5 graphically represents a selection of some of the most interesting enrichment results. In particular, it shows some potential pathways, and their connections, in which ESRP1 may be involved, through our proteomics data and their links with proteins found from the STRING database, and warrants further studies.

## 4. Materials and Methods

### 4.1. CRC Cell Lines, Modulation of ESRP1 Expression

The CRC cell lines used in this study were tested and authenticated, and characterized by genetic and transcriptional profiling, as we previously reported [37]. COLO320DM cells were grown in RPMI (Invitrogen), 10% Fetal Bovine Serum (FBS) and penicillin/streptomycin (PS), while HCA24 was cultured in DMEM, 10% FBS and PS. Caco-2 cells were cultured as previously described [2]. ESRP1 knockdown and overexpression were performed using PLKO.1 lentivirus harbouring ShRNA against ESRP1 or pLX304 lentivirus containing human ESRP1 ORF are as previously described [2].

### 4.2. Chemicals and Reagents

Urea, protease inhibitors, ammonium persulfate (APS), bromophenol blue, glycerol, N,N,N′,N′-tetramethylethylene-diamine (TEMED), sodium dodecyl sulfate (SDS), TRIZMA, 3-[(3-cholamidopropyl) dimethylammonio]-1-propanesulphonate (CHAPS), dithiothreitol (DTT), iodoacetamide, ethanol, ammonium sulfate, phosphoric acid, Coomassie G-250 were purchased from Sigma-Aldrich (St. Louis, MO, USA). DC Protein assay kit, acrylamide, agarose, ready-made immobilized pH gradient (IPG) strip were purchased from Bio-Rad (Hercules, CA, USA). Ampholine at pH 3.5–10 and 5–8 were obtained from GE Healthcare (Milan, Italy).

### 4.3. RNA Extraction and Real-Time PCR

RNA was extracted using the PureLink RNA kit (Ambion, ThermoFisher Scientific, Rodano, Italy) and cDNA prepared using the High-Capacity cDNA Reverse Transcription Kit (Applied Biosystems, ThermoFisher Scientific, Rodano, Italy). Target gene expression was analyzed by Real-time PCR (qRT-PCR) (Supplementary Materials) and normalized to endogenous 18 s expression as previously described [2]. 

### 4.4. RNA-Immunoprecipitation

Total cell protein extracts were obtained by incubating HCA24 cells for 5 min in cold isotonic buffer (20 mM HEPES,100 mM NaCl, 250 mM Sucrose, 5 mM MgCl2, a cocktail of protease inhibitors (Roche, Milan, Italy) and RNAse inhibitor (Promega, Milan, Italy) and DTT. The lysates were precleared for 1 h at 4 °C using sepharose protein A beads. Anti-ESRP1 antibody (Sigma-Aldrich, Milan, Italy) or rabbit IgG was added to the precleared lysates overnight at 4 °C and the day after, sepharose A beads were added for 3 h at 4 °C. After washing, the beads-bound proteins were processed for western blot or proteomics assay.

### 4.5. 2D Gel Electrophoresis and Mass Spectrometry Analysis

Cells were resuspended in 120 µL of a solution containing 9M urea, 4% *w*/*v* CHAPS, 1 mM ortovanadate, 80 mM DTT, protease inhibitors and nuclease. Sample preparation, first and second dimensional separations were performed essentially as previously described [38]. 2D gel electrophoresis (2-DE) was carried out by loading 200 µg of protein for each sample onto ready-made IPG strip (7-cm IPG strips, pH 3-10NL). Electrophoresis was performed on 12% acrylamide gels. Gels were stained with colloidal Coomassie (18% *v*/*v* ethanol, 15% *w*/*v* ammonium sulfate, 2% *v*/*v* phosphoric acid, 0.2% *w*/*v* Coomassie G-250) for 48 h. 2-DE images were analyzed using PD-Quest software (version 7.2, Bio-Rad Laboratories, Segrate, Italy) according to the manufacturer’s instructions. Normalization of each individual spot was performed according to the total quantity of the valid spots in each gel, after subtraction of background values. The spot volume was used as the analysis parameter to quantify protein expression. Experiments were performed in triplicate. A two-sided Student’s *t*-test was used to verify the significance of the variations of expression. Statistical significance was set at *p* values ≤ 0.05. Protein digestion, mass spectrometry analysis and database search were performed as previously described [38]. Due to the small number of proteins identified by this analysis, a Mascot score of ≥50 was considered significant.

### 4.6. Western Blotting

Proteins were extracted and SDS-PAGE (Biorad) performed as previously described [2]. Antibodies used are described in Supplementary Materials. Densitometric analysis was performed using Image Lab software (Bio-Rad Laboratories, Segrate, Italy).

### 4.7. Bioinformatics Analysis

#### 4.7.1. Protein-Protein Interaction

Database STRING version 11 has been used to study protein-protein interaction [39,40]. In particular, protein-protein pairs with a combined score higher than 700 have been retained for analyses. For each protein found experimentally linked to ESRP1 and for ESRP1 itself, we isolated a Ranked Coexpression Set (RCS) defined by the seed protein together with the *n* proteins most closely associated with it, and thus contained *n + 1* objects, *n* depending on the database mined. In particular, our RCSs were composed of 15 proteins (determined by requesting the same number of related proteins for all RCSs and by imposing a minimum of 10 linked proteins) with the highest STRING combined score. The same number of objects for every RCSs guaranteed the same statistical power for every protein in the enrichment analysis subsequently performed. We isolated 26 RCSs of proteins and lost some sets because of the small number of associated proteins.

#### 4.7.2. Enrichment Analyses

Bioconductor package *ClusterProfiler* has been used for enrichment analysis [41]. We focused on Biological Process, Cellular Component and Molecular Function of Gene Ontology (GO) resource, Hallmark gene sets of Gene Set Enrichment Analysis (GSEA) from MSigDB Collections (version 6.2) [42]. GO keywords with no more than 2000 associated genes were analysed and Benjamini Hochberg (BH) strategy for False Discovery Rate (FDR) was applied by using a cutoff at 0.05 both for *p* value and *q*-value.

#### 4.7.3. Proteomics Data and Correlation Analysis

Proteomics data were obtained from “*Colorectal Cancer Cell Line Proteomes Are Representative of Primary Tumors and Predict Drug Sensitivity*” by Wang et al. [31]. In particular, spectral count data for 95 tumor samples derived from CPTAC COADREAD cohort were used. Raw counts were normalized by diving the count data by the total number of counts for each sample and then log-transformed in base 2. R cor.test function was used to evaluate Spearman correlation coefficient.

### 4.8. Statistical Analyses

Data in bar graphs are expressed as mean ± standard deviation. Statistical differences were determined by a 2-tailed Student’s *t*-test (* *p* < 0.05, ** *p* < 0.01, *** *p* < 0.001).

## 5. Conclusions

Our data provide clues into the complex functional modules associated to ESRP1 in CRC (depicted in Figure 5) and reveal several new proteins modulated by or bound to ESRP1 in support of its pro-oncogenic role in CRC. The integrative proteomics-bioinformatics approach we employed unveils several potential oncogenic pathways activated by ESRP1 in this tumor. Understanding the function of RBPs and their regulation and interaction network with high-throughput techniques as well as integral analysis of multiple datasets in cancer cells will help in developing prognostic and response biomarkers and potentially unveil new targets for the design of therapeutics.

## Supplementary Materials

Supplementary materials can be found at https://www.mdpi.com/1422-0067/21/2/575/s1.

## Abbreviations

ESRP1	Epithelial Splicing Regulatory Protein 1
CRC	ColoRectal Cancer
RIP	RNA Immuno Precipitation
RBP	RNA-Binding Protein
PPI	Protein-Protein Interaction
EMT	Epithelial to Mesenchymal Transition
GO	Gene Ontology
Scr	Scrambled
Sh	Short hairpin
MOI	Multiplicity of Infection
2-DE	2-dimensional gel electrophoresis
RCS	Ranked Correlated Sets
