# Supplementary material for: Proteomics-Based Evidence for a Pro-Oncogenic Role of ESRP1 in Human Colorectal Cancer Cells
Source: Int J Mol Sci. 2020 Jan 16;21(2):575. doi: 10.3390/ijms21020575 (PMC7014300; doi:10.3390/ijms21020575)
Supplement: Supplementary file 1 [file ijms-21-00575-s001.zip › Supplementary Table 3.pdf]

Supplementary Table S1: Primers used for Real-time PCR

qRT-PCR Primers name	5'-3' sequence	UPL probe
SF3A1_Left	tcatgccagaggaggagttc	#47
SF3A1_Right	ttgggcacctggactttg	
FBF1_Left	gaggcacggctgaatgag	#15
FBF1_Right	actccgccttggactgct	
TPI1_Left	gttgggggaaactggaagat	#38
TPI1_Right	tagggggagcacaaaccac	
CCAR2 left	tcatggacggttggatccag	#22
CCAR2 right	atgccttggcttcttagcac	
ESRP1 left	tgatcttcgaaaagaattcaagaaa	#37
ESRP1 right	cgagagactgaactactcttctcaaaa	
