# Supplementary material for: Proteomics-Based Evidence for a Pro-Oncogenic Role of ESRP1 in Human Colorectal Cancer Cells
Source: Int J Mol Sci. 2020 Jan 16;21(2):575. doi: 10.3390/ijms21020575 (PMC7014300; doi:10.3390/ijms21020575)
Supplement: Supplementary file 1 [file ijms-21-00575-s001.zip › Supplementary Table 4.pdf]

Supplementary Table S2: Antibodies used

Antibodies	Company
ESRP1	Sigma
ESRP1	Abcam
SF3A1	Thermo Scientific
CCAR2	Sigma
HSP90	Sigma
FBF1	ProteinTech
TPI1	ProteinTech
