# Supplementary figures and images for: Proteomics-Based Evidence for a Pro-Oncogenic Role of ESRP1 in Human Colorectal Cancer Cells
Source: Int J Mol Sci. 2020 Jan 16;21(2):575. doi: 10.3390/ijms21020575 (PMC7014300; doi:10.3390/ijms21020575)

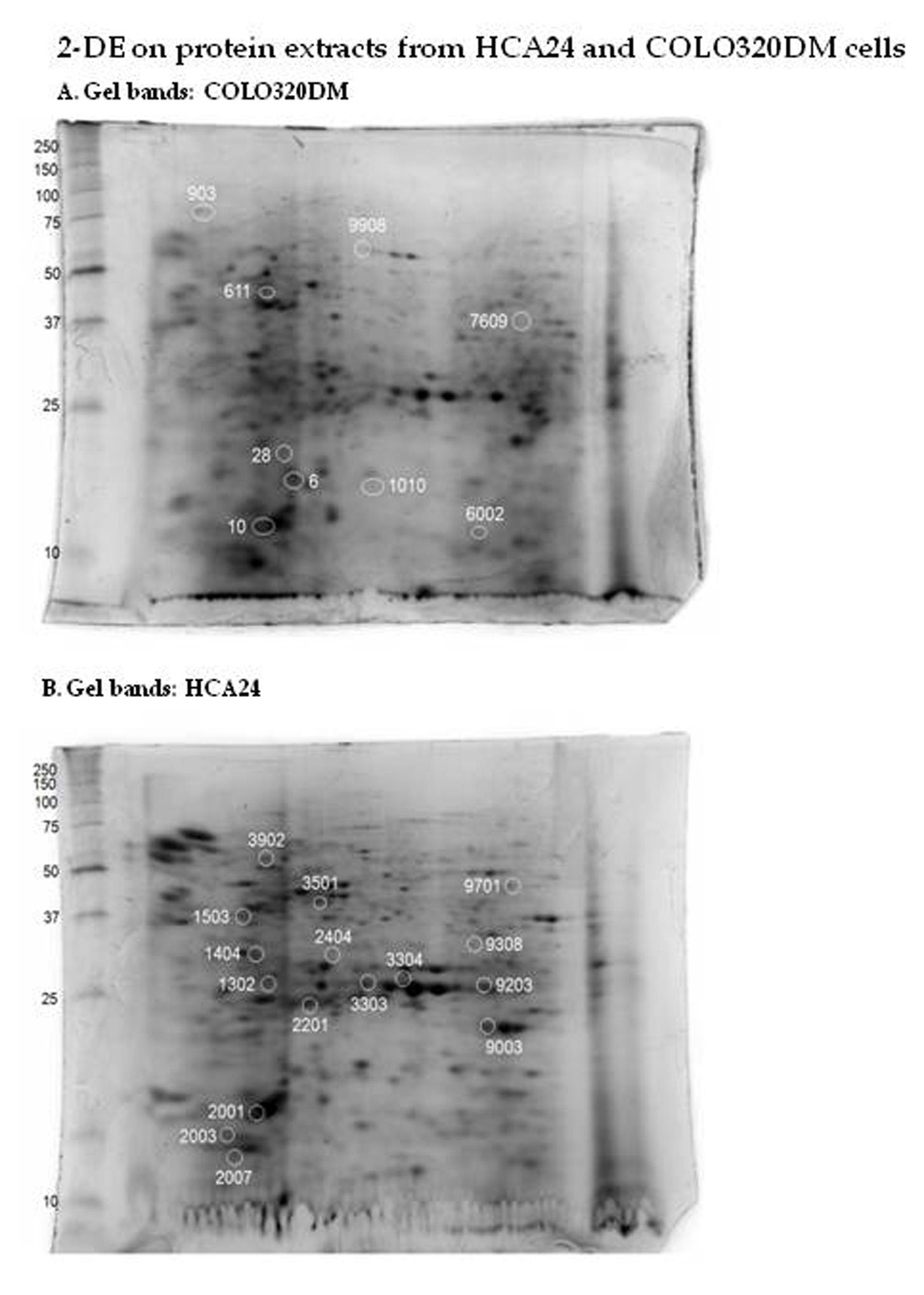

Supplement: Supplementary file 1 [file ijms-21-00575-s001.zip › Supplementary Figure 1.JPG]

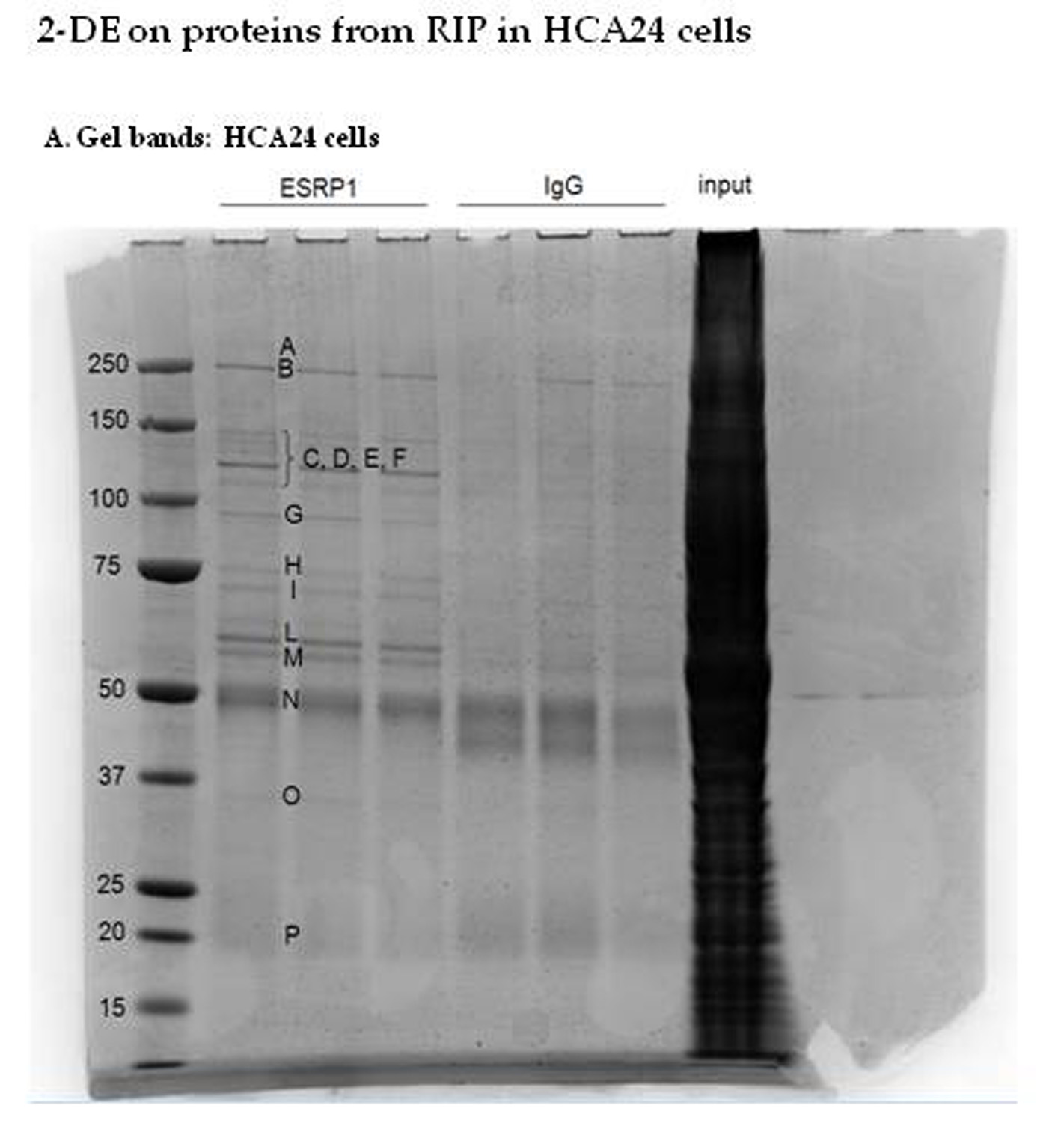

Supplement: Supplementary file 1 [file ijms-21-00575-s001.zip › Supplementary Figure 2.JPG]

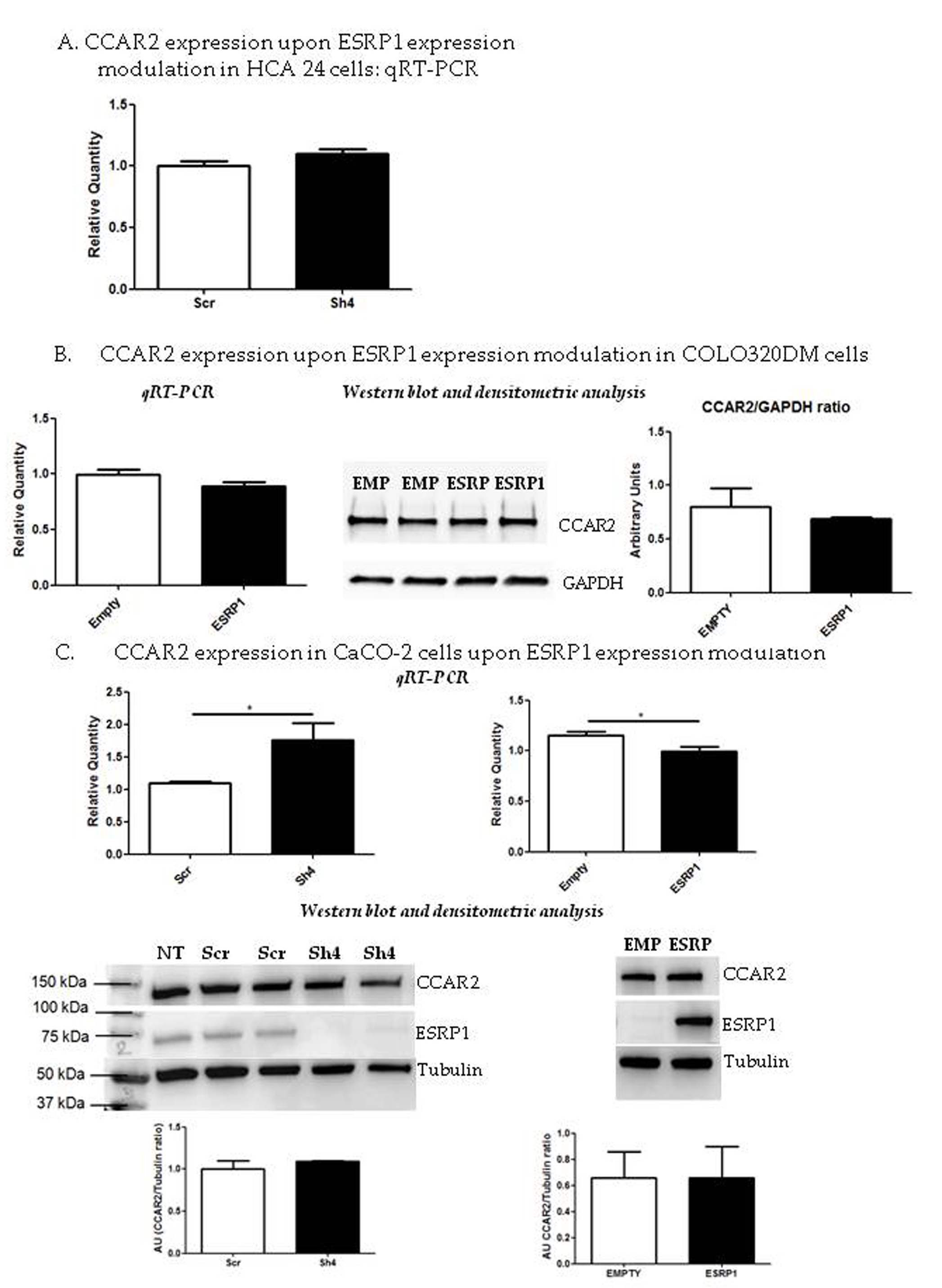

Supplement: Supplementary file 1 [file ijms-21-00575-s001.zip › Supplementary Figure 3.jpg]

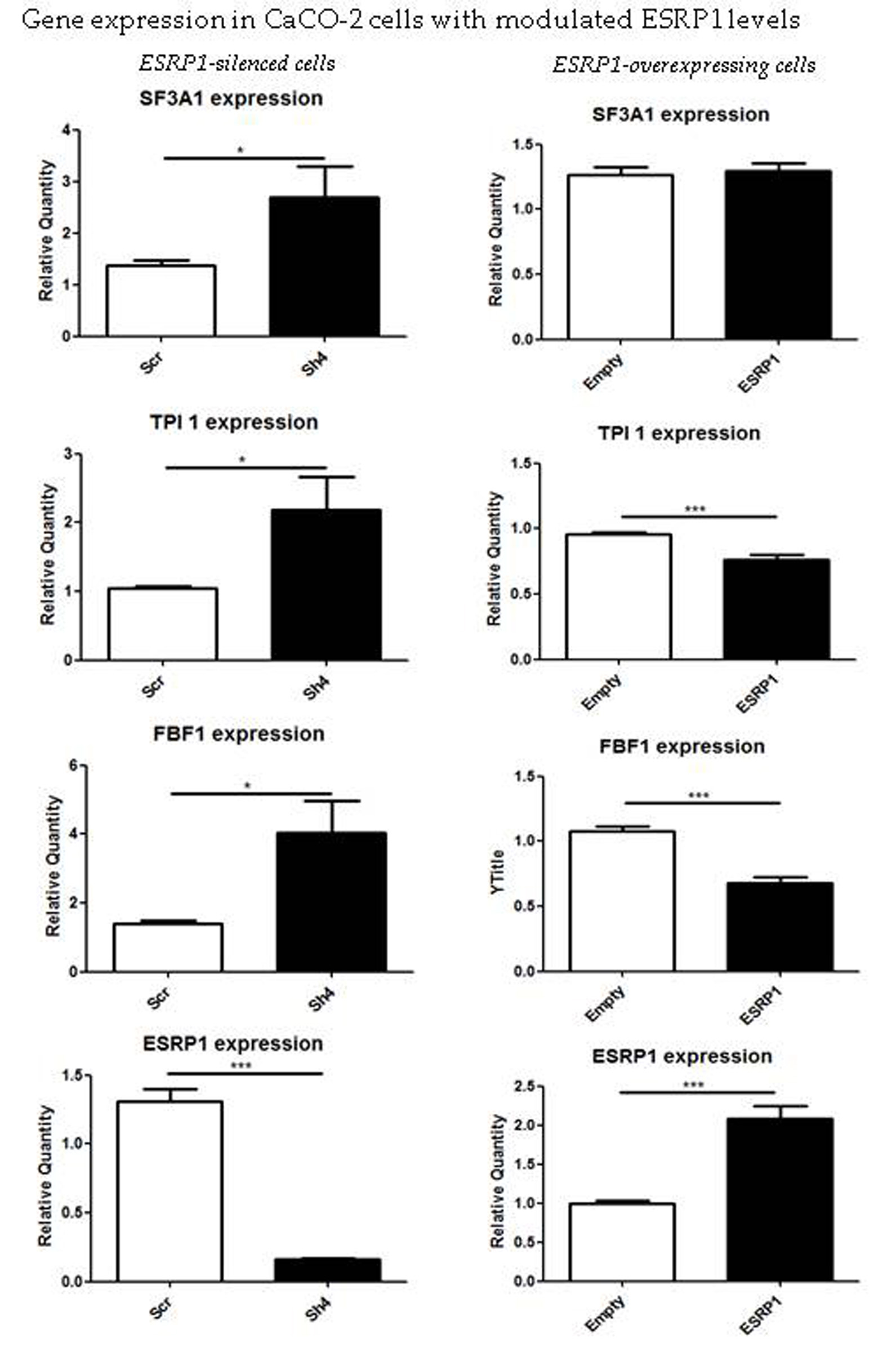

Supplement: Supplementary file 1 [file ijms-21-00575-s001.zip › Supplementary Figure 4.JPG]
